# Fish Lymphocytes: An Evolutionary Equivalent of Mammalian Innate-Like Lymphocytes?

**DOI:** 10.3389/fimmu.2018.00971

**Published:** 2018-05-07

**Authors:** Giuseppe Scapigliati, Anna M. Fausto, Simona Picchietti

**Affiliations:** Dipartimento per l’Innovazione nei sistemi biologici, agroalimentari e forestali, Università degli Studi della Tuscia, Viterbo, Italy

**Keywords:** innate immunity, innate-like lymphocytes, fish lymphocytes, innate lymphoid cells, comparative immunology

## Abstract

Lymphocytes are the responsible of adaptive responses, as they are classically described, but evidence shows that subpopulations of mammalian lymphocytes may behave as innate-like cells, engaging non-self rapidly and without antigen presentation. The innate-like lymphocytes of mammals have been mainly identified as γδT cells and B1-B cells, exert their activities principally in mucosal tissues, may be involved in human pathologies and their functions and tissue(s) of origin are not fully understood. Due to similarities in the morphology and immunobiology of immune system between fish and mammals, and to the uniqueness of having free-living larval stages where the development can be precisely monitored and engineered, teleost fish are proposed as an experimental model to investigate human immunity. However, the homology between fish lymphocytes and mammalian innate-like lymphocytes is an issue poorly considered in comparative immunology. Increasing experimental evidence suggests that fish lymphocytes could have developmental, morphological, and functional features in common with innate-like lymphocytes of mammals. Despite such similarities, information on possible links between conventional fish lymphocytes and mammalian innate-like lymphocytes is missing. The aim of this review is to summarize and describe available findings about the similarities between fish lymphocytes and mammalian innate-like lymphocytes, supporting the hypothesis that mammalian γδT cells and B1-B cells could be evolutionarily related to fish lymphocytes.

## Introduction

Vertebrate-type adaptive responses with MHC, RAG, memory, are present in only 2% of metazoans, but invertebrates can live very long protected by their innate immune defenses. Indeed, invertebrates classically defined as relying only on innate responses may live for centuries and have been found to respond to reinfection, suggesting that innate immunity mechanisms need more investigation. In a comparative immunology view, it is conceivable to speculate that leukocytes populations that emerged early in vertebrates evolution inherited and retained some invertebrate features related to antigen recognition and elimination. During evolution, genes coding for immune activities accumulated toward mammals in a form of “layers.” This hypothesis proposes that evolution produced a layered immune system in which following descendants obtain predominance during development, giving rise to cell populations responsible for progressively more complex immune activities. As it is commonly thought that “ontogeny resembles phylogeny,” a “layered immune system” hypothesis may give clues to understand cell functionality in vertebrates and provide knowledge for a better understanding of human pathologies.

Immune innate responses are a first-level of protection against infection and damage, exerted by cells reacting fast to non-self/injury with their germline-encoded receptors. In mammals, there are different types of innate immune cells, besides macrophages/dendritic cells/neutrophils, also innate lymphoid cells (ILC) have been described. ILC are classified in three groups for the expression of defined transcription factors, functional characteristics, and phenotype. Another group of mammalian unconventional or innate-like lymphocytes (mILL) has been identified with properties and functions as a bridge between innate and adaptive responses. Populations of mILL might thus rerepresent an “immune lower layer,” with activities involved in maintaining gut homeostasis, in early response to intestinal infections, in autoimmune diseases and cancer, in a fast unprimed fight against infection and damage, in producing germline natural polyreactive antibodies and typical cytokine patterns. The mILL have been mainly identified as γδT cells and B1-B cells, are mainly located in mucosal tissues, and their functions and origin are still under scrutiny.

Of note, increasing evidence suggests that conventional fish lymphocytes display some developmental, morphological, and functional features in common with mILL and, very recently, these similarities have attracted attention among immunologists (1). However, studies aimed to clarify the links between fish lymphocytes and mIL are at their infancy.

This review is proposed to summarize the current knowledge on possible similarities between fish lymphocytes and mILL, and use the knowledge to raise the hypothesis that most of fish lymphocytes behave like subpopulations of mILL and, consequently, that mILL subpopulations (γδT cells, B1-B cells) could represent a “lower layer” of extant, evolutionary-related, analogs of fish lymphocytes.

## Mammalian Innate-Like Lymphocytes

Important players of innate immune activities are the mammalian ILC that derive from a common lymphoid precursor and play a role with effector and regulatory functions in innate immunity and tissue remodeling. The ILC do not have TcR or Ig rearranged receptors in their surface and are classified into three groups on the base of the patterns of cytokines they produce, and of transcription factors necessary to their functions. Namely, ILC1 produce IFNγ and depend on Tbet, ILC2 produce type 2 cytokines (IL-5/IL-13) and require GATA3, ILC3 depend on RORγt and produce IL-17 and/or IL-22 (2). Also natural killer (NK) cells belong to innate lymphocytes are involved in fast innate responses and do not express CD3 or lymphocyte receptors on their surface. However, aside from the classical description of lymphocytes as cells responsible of adaptive responses, subpopulations of recently discovered mILL behave as innate immune cells with respect to the historical innate-adaptive classification (3).

The mILL are involved in maintaining gut homeostasis and in early response to intestinal infections (4, 5), in autoimmune diseases and cancer (6, 7), are able to combat non-self in an MHC-independent fashion (8, 9), produce unbiased natural polyreactive antibodies (10, 11) and typical cytokine patterns (12, 13).

The main lymphocyte subpopulations displaying innate-like activities in mammals have been identified as γδT cells (5), mucosa-associated invariant T cells (MAIT) (14), natural killer T cells (NKT) (15), B1-B cells (16), and spleen marginal zone B cells (17).

### Innate-Like T Cells

The γδT lymphocytes are non-conventional T lymphocytes, comprising a minor T cell subset in blood and a major population of intestinal intraepithelial lymphocytes (IELs) having typical morphological features of lymphocytes with a surface germline TCR phenotype of γ^+^δ^+^ (mostly displaying repertoires Vδ1/Cγ1 and Vγ9/Vδ2) and showing a potent phagocytic ability to both soluble and particulate antigens (18, 19). With respect to immunoglobulins and αβTcR molecules, the γδTcR displays the highest spontaneous diversity in the CDR3 region produced by VDJ recombination by using the V-chain gene. The γδT cells can develop extrathymically and independently from an antigen encounter and are active players in adaptive and innate-like immune responses such as the direct killing of infected cells, are involved in tumor immunosurveillance (20), produce molecules required for pathogen clearance (21), are spontaneously cytotoxic (22), release immunomodulatory cytokines (23), and can be activated by stress-induced molecules (MIC-A/B, ULBPs) to produce pro-inflammatory cytokines and lytic enzymes. In summary, evidence suggests that γδT cells act either as effectors and regulators (24), and represent an evolutionarily primitive T cell subset characterized by innate and adaptive immune functions. Supporting these findings, recent data also showed the presence of γδT cells subsets for which innate stimuli are more important than TcR ligation, as in the case of IL-17-producing (γδT-17) and IFNγ-producing (γδT-IFNγ) cells (25).

Other subpopulations of recently discovered mammalian innate-like T lymphocytes are the MAIT and NKT. MAIT are an innate T cell subpopulation (14), principally involved in antibacterial immunity at mucosal surfaces, and mainly present in man than in mouse (26), they display a germline TcRαβ phenotype (Vα7.2-Jα33/12/20 in humans, Vα19-Jα33 in mice) and variable but restricted TcRβ chains (5, 27). Upon stimulation, MAIT produce the regulatory cytokines IFNγ, TNFα, and IL-17, and express the receptors for IL-7, IL-12, and IL-18 (26).

The NKT are a subpopulation of αβ- and γδ-T cells differing from NK cells for the presence of CD3 and TcR, characterized by CD1d restriction and limited TcR diversity (15, 28). They are principally present in non-mucosal tissues, are involved in antitumor activity, and are of help for B cell proliferation and antibody production (29). The NKT can be further divided into two distinct subpopulations, namely, type I and type II NKT cells (30) that are preferentially located in the liver. Type I display a semi-invariant TcR (Vα14Jα18/Vβ2, 7, 8) in mice and (Vα24Jα18/Vβ11) in humans, whereas type II NKT cells exhibit a more diverse TcR repertoire.

### Innate-Like B Cells

The B lymphocytes of mammals are now cataloged as B2, or classic, and B1, or innate. These two major sets of B cells are defined by differential presence of CD5 in their surface. The B1-B cells are further subdivided in B1a (B1) having a phenotype CD5^+^/IgM^high^/IgD^low^, and B-1b cells, which are CD5-negative (31). The B1-B cells produce large amounts of natural polyreactive antibody in a T cell-independent manner, are actively phagocytic and microbicidal (32), may be involved in autoimmunity (33), and are present as IgA-secreting plasma cells in the intestinal mucosa where they migrate during infections (16). Natural polyreactive antibodies produced by CD5^+^ B cells are germline-encoded antigen recognition molecules (class IgM, IgA, and IgG3) (11) with a limited repertoire of V-region genes, play an important role in early host defense, in autophagy/tissue remodeling and immune regulation, in recognition of pathogens and activation of the innate immune system *via* the classical pathway of complement activation (10). The B1-B cells are considered to have no memory, are present in mouse liver at fetal stages (34), whereas in adults are present in the spleen and peritoneal cavity (35, 36), where they undergo self-renewal with mechanisms that are poorly understood.

Being involved in innate activities, B1-B cells respond to stimulation *in vitro* through TLRs (from TLR1 to TLR8) (37, 38) inducing B1-B cell proliferation and differentiation into immunoglobulin-secreting cells. Also, B1-B cells show a rapid capacity to produce high amounts of the immunomodulatory cytokine IL-10 after innate activation (13).

An additional subpopulation of B cells having innate-like activities is located in the spleen pulp marginal zone and involved in producing IgM antibodies in a T cell-independent manner against pathogens circulating in blood (17).

Of particular interest is the tissue localization of innate-like B cells, which exert their activities principally in mucosal surfaces and mainly in the intestine, where the IgA produced by B1-plasma cells can be spontaneously present, reacting with the intestinal microflora (39). The mucosal intestine is also the richest site of γδT lymphocytes in adult mice and man (40), followed by the respiratory epithelium (24), and the epidermis (41). In mucosal tissues, during a possible infection the mILL displaying germline receptors can respond quickly, thus providing protection independently from adaptive responses and in the absence of antigen exposure as, for instance, in newborns (5).

## Fish Lymphocytes

The features of mILL, very briefly summarized above, appear to be remarkably similar to the features of conventional lymphocytes as they are known in teleost fish, where experimental data accumulated in decades of investigation showed the presence of T cells possessing surface αβ- and γδ-TcR, of B cells expressing three immunoglobulin types (IgM, IgT, and IgD), of lymphocyte subpopulations, and a complete set of master genes coding for lymphocyte-associated molecules (42–45). The fish lymphocytes have been shown to be functionally active *in vitro* and *in vivo* (46–52), and to produce and/or be affected by families of lymphocyte-related cytokines (53, 54).

### Features of Fish T Cells

Two classes of T cells are present in teleost fish, displaying on their cell surface αβ- and γδ-TcR, together with TcR coreceptors, and expressing patterns of genes that clearly indicate the presence of T cell subpopulations as they are known in mammals, namely, cytotoxic (CD8), helper (CD4), and regulatory (Treg, Th17) (45, 55–57). The immunobiology of fish T cells has been the subject of extensive research addressed to investigate regulation mechanisms, expression of surface markers, and *in vitro/vivo* studies, that have been reassumed in recent reviews (42, 53, 54, 58–60). In relation with the present work, available data have shown that the distribution of T cells in fish is principally located in mucosal tissues of intestine and gills (60–66), and that activities of T cells are diverse in these tissues. In the intestine, IEL displays an *in vitro* spontaneous cytotoxic activity (65), proliferate poorly (unpublished), and perform *in vivo* RAG-driven spontaneous somatic rearrangement of a given V/C combination in the CDR3 junction length of TcRβ-chain/TcRγ-chain in the absence of antigen stimulation (64, 67). On the other hand, T cells from the gills are able to proliferate *in vitro* in response to lectins, but RAG expression is negligible (45). These observations suggest that the teleost intestine could be a site of production of T cells, whereas the gills could be a site where T cells are more committed as effectors/helper. A support to the hypothesis that the fish intestine can be a primary producer of T cells comes from data on the development of sea bass immune system, where first antibody-positive T cells are detected in the developing gut before, or at the same time, than in thymus (68, 69). However, definitive knowledge establishing precise timing and tissue of appearance of T cell subpopulations in fish is still missing (70).

The intestine of sea bass displays a high homogeneous expression of TcRα and TcRγ, a low expression of CD4, and differential expression of CD8α and of MHC_II_ showing an increment and a decrease, respectively, toward the terminal part (65). Considering the number of T cells present in the intestinal mucosa, and that purified T cells from the intestine with a pan-T mAb showed enriched expression of RAG-1, TcRα, TcRγ, CD8α, and CD4, it appears evident that the gut can be considered the main lymphoid tissue for T cells in adult fish (43).

Data obtained on *in vitro* activity of fish leukocytes suggest the presence of an IL-2 modulated proliferation of T cells during a mixed-leukocyte reaction (48) and of a MHC-restricted CTL activity (52), suggesting that fish immune cells also display activities comparable to classical T lymphocytes of mammals.

Finally, fish do have memory T cells, identified by the IL-10 modulation of CD8- and CD4-populations responses and proliferation in immunized carp (71). Interestingly, it should be noted that mutant zebrafish engineered for lacking somatic recombination (RAG-1^−/−^) are still able to mount a specific protection after bacterial re-exposure (72), and that Atlantic cod lacks CD4 and MHC_II_ in the genome but is protected during immune challenges with pathogens (73). These latter observations suggest that further research is needed in fish to better elucidate functional features of T cells, such as the phagocytic capability of γδT cells (18).

### Features of Fish B Cells

Production of antigen-specific antibody in fish is known since almost 70 years, and research has shown that fish have B cells expressing three heavy Ig chain classes, namely, IgM, IgT/Z, and IgD, as defined by the expressed genes μ, τ, and δ, respectively (74–76), and of some Ig light chains (e.g., two in catfish, three in zebrafish, MW 25–28 kDa) (77). The IgM are tetrameric in fish (MW 450 kDa) and present systemically in body fluids, where they may be present in serum at high concentration. The IgT/IgZ are mucosal immunoglobulins produced in a monomeric form (MW 170 kDa), although a non-covalent polymeric IgT association has been observed in trout mucus. The IgD has been studied at molecular level, it is expressed in a monomeric form with a putative MW of 150 kDa, but little is known on its physiological role in fish (78). Likewise T cells, the B cells of fish have been the subject of much research, with results reassumed in comprehensive reviews (44, 79, 80). With respect to the present work, main activities of fish B cells can be summarized as follows: (i) high content of natural serum IgM in unimmunized fish (81–83); (ii) poor increase in IgM affinity after secondary immunization (84, 85); (iii) presence of memory B cells (85); (iv) spontaneous phagocytosis (86); (v) production of pathogen-induced mucosal secretory IgT (evolutionary orthologs of IgA) (49); (vi) presence of kidney lymphocyte precursors similar to mouse spleen B1-B cells (34, 87); and (vii) presence of proliferating B cells in the peritoneal cavity (88); possible expression of TLRs (89). Interestingly, intriguing features regarding B cells have been observed in some fish species as, for instance, the lacking of pathogen-specific IgM in gadoids after successful immunization against the pathogen (82), and a lack of the whole IgM gene in a coelacanth species (90).

### Similarities Between Mammalian Innate-Like and Fish Lymphocytes

**Figure 1:**
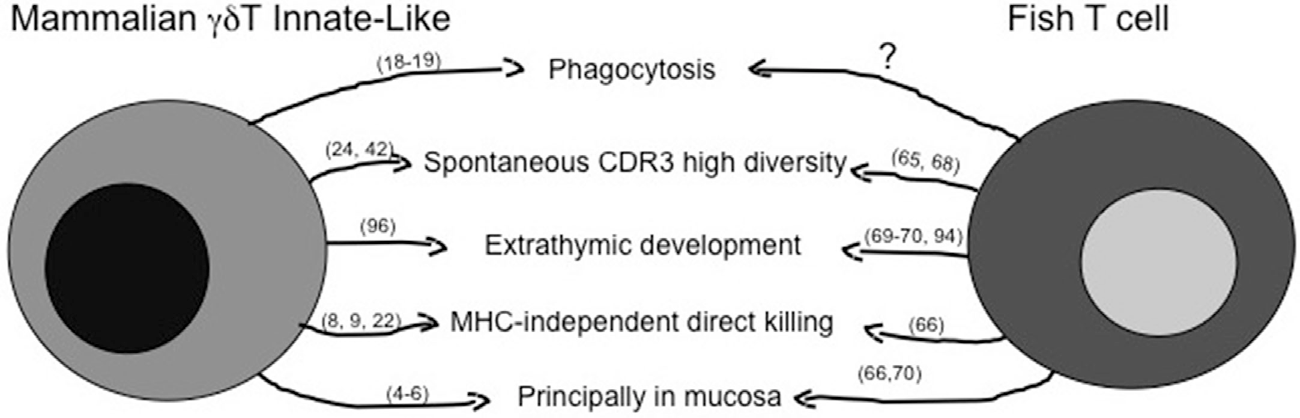
Similarities between mammalian innate-like T lymphocytes and fish T cells. The figure reassumes similar immune activities of γδT innate-like T cells as they are known in mammals and in fish. The question mark represents activities likely present, but not fully demonstrated yet. Main references for the indicated activities are reported in brackets.

**Figure 2:**
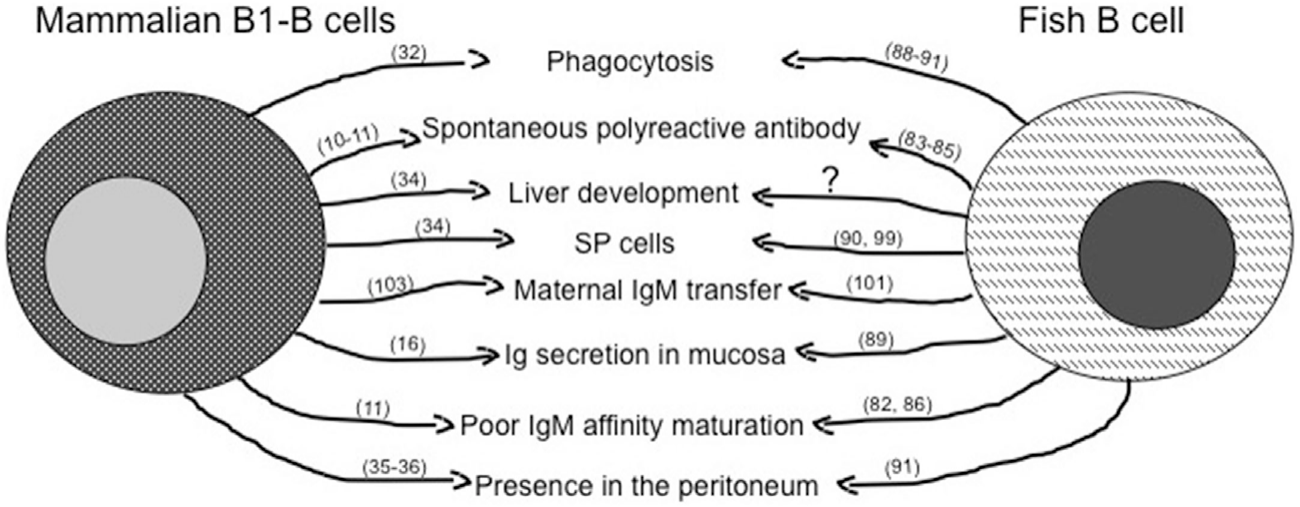
Similarities between mammalian innate-like B lymphocytes and fish B cells. The figure reassumes similar immune activities of B1-B cells as they are known in mammals and in fish. The question mark represents activities likely present, but not fully demonstrated yet. Main references for the indicated activities are reported in brackets.

The principal features of mILL and of fish conventional lymphocytes have been briefly summarized above, and a comparison of possible similarities is shown in Figure 1 for T cells and in Figure 2 for B cells. A point of great importance to better understand the evolution of lymphocytes among vertebrates is the definition of the primary tissue(s) of origin and of tissue localization during development. Experimental evidence suggests that the fish intestine may be a primary lymphoid tissue for T cells, which can be detected there even before their appearance in thymus (68, 91–93), and where a T cell selection might be present that differs from thymic T cell selection. The possible thymus-independent origin of T cell subpopulations appears to be conserved until mammals, where γδT cells may derive from human fetal liver and the primitive intestine between 6 and 9 weeks of gestation, as proposed by investigating expression of the δTcR repertoire during human development (94, 95). Indeed, γδT cells play a pivotal role during human intestine development, since preterm infants with intestinal barrier immaturity, and thus with a reduction in the number of IEL, may develop severe enterocolitis (96).

The importance of liver as a site of possible lymphocytes development emerges from data on the origin of B cells in mammals, where B1a cells have been found to develop in mouse fetal liver, from which they migrate in the spleen, but not in the bone marrow (34). Interestingly, in the mouse spleen, a lymphocyte subpopulation shows flow cytometric morphological features (SP cells) remarkably similar to that of lymphoid SP cells from adult goldfish and zebrafish kidney (87, 97).

The origin of B cells in fish is not clearly defined, in zebrafish the pancreas has been supposed to be a primary site on the base of B cell receptors genes rearrangement (98), whereas in the sea bass kidney a presence of IgM-producing cells has been established by IHC at 55 days post hatching (69). Although it is evident in all fish species investigated that the development of T cells precedes development of B cells, a definitive clarification of a primary site of B cell origin is missing (69).

Another similarity could be found in the transmission of immunity between the female and the developing embryo. In fish, a possible precursor process of the maternal antibody transfer to the fetus through placenta has been observed by the presence of IgM molecules and IgM gene expression in unfertilized eggs and during first embryonic stages (99). In mammals, the B1-B cells are already present at early stages in the extraembryonic yolk sac and continue their development in the fetal liver (100), with the IgM being the predominant class during late gestation and infancy (101).

Other experimental evidence suggests striking similarities between low affinity polyreactive serum natural IgM antibodies produced by mammalian B1-B cells (13) with IgM presence/responses in fish (80). As in mammals and other investigated vertebrate species, the kinetic of primary antibody response in fish involves IgM but, at variance with mammals, in fish there is neither a class-switch secondary response nor a substantial increase in serum IgM affinity, although specific antibody titers can be observed after immunization. Of note, the protection mechanisms and specificity of antibody responses in fish are far to be fully understood, since some fish species result protected after immunization without producing specific IgM antibody (82). In addition, the IgM can be even totally absent, as discovered in a species lacking completely of IgM genes (90). The possible importance of natural IgM in fish as players in innate immunity emerges from their amount in serum, since the mean concentration of IgM in unimmunized fish (sera from five species, 7.7 mg/ml) (81) is much higher than the mean concentration of IgM in humans (1.3 mg/ml) (102). Considering that fish lack IgG, the higher concentration of natural IgM could contribute to immunity against pathogens in not yet completely understood ways, suggesting that research on natural IgM contribution in innate immunity, and the kinetic of production of specific IgM by B cells upon immunization, may give some clues to understand the physiology of natural antibodies in mammals.

The Ig secreted in/by mucosal tissues are particularly important for pathogen clearance at the boundary with external environment, and teleost fish have a mucosa-associated IgT class whose features, like the coating of intestinal commensal microbiota, precede that of mammalian-specific secretory IgA (49). Although IgT is not homologous to IgA, it is evident a convergent evolution of the two molecules, both are multimeric, predominantly produced in the mucosa, and induced by mucosal immunization (103).

Fish leukocytes express TLRs (90), show strong *in vitro* response to LPS, and respond to flagellin with TLR5 (104), and to viruses and poly I:C with TLR3 (105). Although these responses have been measured in leukocytes, it should be reasonable to speculate that fish B cells should express pathogen-specific conserved TLRs on the base of nucleotide sequences obtained from a transcriptome of head kidney, a B cell lymphopoietic tissue in fish that revealed the presence of several TLRs’ gene expression (106). Given the presence of TLRs on fish B cells, a similarity becomes evident with TLRs on mammalian B1-B cells (37, 38).

Another population of fish IgM-B cells is located in the peritoneal cavity, capable of proliferate very soon after antigenic stimulation, produces polyreative antibodies, and is responsible of pathogen clearance (88). Similarly, in mammals the peritoneal B1-B cells can proliferate rapidly after antigen stimulation and can migrate in the periphery, including the intestine, to fight the pathogen (107).

## Summary

The immune defense system of vertebrates in its molecular and cellular components is remarkably conserved from teleost fish, the more ancient extant representatives of the evolutive lineage that directly brings to mammals. The knowledge on the similarities between morphological and physiological processes of vertebrates led to the use of teleost fish as an additional animal model for investigations in pathology and physiology of immune recognition, with the goal of applying results in translational research for modeling human diseases, as can be easily appreciated with the zebrafish model. Therefore, teleost fish play a fundamental role in understanding the evolution of immune responses of vertebrates, and experimental evidence suggests that some features of mammalian innate-like lymphocytes related to pathogenic conditions, such as chronic lymphocytic leukemia and inflammation could benefit from knowledge in fish lymphocytes.

The hypothesis described in this review is that younger species (mammals) retain immune defense features of ancestors (fish) that have been enriched by evolution with new “layers” of genes coding for cells and molecules, with a “lower” immune layer that in mammals might be composed of cells with innate activities, among which innate-like lymphocytes.

The experimental evidence considered in this review suggests similarities in morphology, gene expression, and functional signatures of fish lymphocytes with mammalian innate-like lymphocyte subpopulations, although much remains to be learned on the immunobiology of fish lymphocytes such as the origin/functions of intestinal T cells/γδT cells and of B1-B cells.

Considering that MAIT are restricted to mammals, it remains to elucidate the possible presence of NKT in fish, where a clear surface phenotype identification of spontaneously cytotoxic T cells is missing. It also remains to investigate in more details in fish a precise timing and tissues of origin of αβ/γδT cells, of IgM/IgT B cells, their transcriptomic signatures, and some functional activities like production and kinetic properties of natural polyspecific IgM and phagocytic capability of γδT cells.

Importantly, the hypothesis that subpopulations of mammalian innate-like lymphocytes, namely, γδT cells and B1-B cells could be an extant-like counterpart of fish lymphocytes has been already proposed (32) and supported by a recent publication (1). These works suggest that the origin of lymphocytes, possibly including innate-like lymphocytes, goes back to the origin of all vertebrates (1).

In conclusion, investigations on the development and immunobiology of fish lymphocytes is of great importance in comparative immunology, and possibly important for a better understanding of mammalian innate-like lymphocytes immunobiology and their involvement in human diseases.

## Author Contributions

GS was responsible of organizing and supervising the review work. AF was responsible for manuscript writing. SP was responsible for checking the literature references and databases and organizes figures.

## Conflict of Interest Statement

The authors declare that the research was conducted in the absence of any commercial or financial relationships that could be construed as a potential conflict of interest.
